# Joint Exposure to Multiple Air Pollutants, Genetic Susceptibility, and Incident Dementia: A Prospective Analysis in the UK Biobank Cohort

**DOI:** 10.3389/ijph.2024.1606868

**Published:** 2024-02-15

**Authors:** Shuo Zhang, Hongyan Cao, Keying Chen, Tongyu Gao, Huashuo Zhao, Chu Zheng, Ting Wang, Ping Zeng, Ke Wang

**Affiliations:** ^1^ Department of Biostatistics, School of Public Health, Xuzhou Medical University, Xuzhou, Jiangsu, China; ^2^ Division of Health Statistics, School of Public Health, Shanxi Medical University, Taiyuan, Shanxi, China; ^3^ Center for Medical Statistics and Data Analysis, Xuzhou Medical University, Xuzhou, Jiangsu, China; ^4^ Key Laboratory of Human Genetics and Environmental Medicine, Xuzhou Medical University, Xuzhou, Jiangsu, China; ^5^ Key Laboratory of Environment and Health, Xuzhou Medical University, Xuzhou, Jiangsu, China; ^6^ Xuzhou Engineering Research Innovation Center of Biological Data Mining and Healthcare Transformation, Xuzhou Medical University, Xuzhou, Jiangsu, China; ^7^ Jiangsu Engineering Research Center of Biological Data Mining and Healthcare Transformation, Xuzhou Medical University, Xuzhou, Jiangsu, China

**Keywords:** multiple air pollutants, long-term exposure, dementia, joint association, prospective cohort study

## Abstract

**Objectives:** This study aimed to evaluate the joint effects of multiple air pollutants including PM_2.5_, PM_10_, NO_2_, and NO_x_ with dementia and examined the modifying effects of genetic susceptibility.

**Methods:** This study included 220,963 UK Biobank participants without dementia at baseline. Weighted air pollution score reflecting the joint exposure to multiple air pollutants were constructed by cross-validation analyses, and inverse-variance weighted meta-analyses were performed to create a pooled effect. The modifying effect of genetic susceptibility on air pollution score was assessed by genetic risk score and *APOE ε4* genotype.

**Results:** The HR (95% CI) of dementia for per interquartile range increase of air pollution score was 1.13 (1.07∼1.18). Compared with the lowest quartile (Q1) of air pollution score, the HR (95% CI) of Q4 was 1.26 (1.13∼1.40) (*P*_trend_ = 2.17 × 10^−5^). Participants with high air pollution score and high genetic susceptibility had higher risk of dementia compared to those with low air pollution score and low genetic susceptibility.

**Conclusion:** Our study provides evidence that joint exposure to multiple air pollutants substantially increases the risk of dementia, especially among individuals with high genetic susceptibility.

## Introduction

Dementia, primarily consisting of Alzheimer’s disease (AD) (which causes 50%∼70% of cases) and vascular dementia (VD) (which causes 25% of cases) [1], is a major public health problem, and places a huge burden on patients, their caregivers and national healthcare systems [2]. Currently, approximately 50 million people worldwide suffer from dementia, with over 10 million new cases occurring each year; this number is expected to increase to 152 million by 2050 [1]. The deaths due to dementia are more than doubled globally from 1990 to 2019, with 1.62 million deaths in 2019, which is the seventh leading cause of deaths [3].

As there is no complete cure for dementia, identifying potential risk factors offers promising opportunities for primary prevention. The development and progression of dementia are associated with genetic and environmental factors [4]. Among environmental determinants, air pollution is an important factor that contributes to incident dementia [5], and a number of epidemiological investigations have uncovered strong association between air pollution and dementia. For example, it has been demonstrated that PM_2.5_ increases the likelihood of occurring dementia [6], and that exposures to PM_10_, PM_2.5_, and NO_2_ are related to higher risk of incident all-cause dementia [7].

However, previous studies mainly focused on evaluating the association between individual air pollutants (e.g., PM_2.5_, PM_10_. NO_2_, or NO_x_) and the risk of dementia [8–10]. It is well-known that ambient air pollution consists of a mixture of particulate and gaseous pollutants, and their joint health effects may be different from the influences of individual air pollutants. To our knowledge, there are currently no prospective cohort studies that examine the joint impact of various air pollutants on the risk of incident dementia.

Furthermore, it has been established that genetics also substantially contribute to incident dementia [11]. For example, three genome-wide association studies (GWAS) published recently have increased the number of dementia-related single-nucleotide polymorphisms (SNPs) to 40 [12–14]. The well-known apolipoprotein *E* gene (*APOE*) and other loci have been identified to be associated with the risk of developing dementia [15]. Moreover, *APOE ε4* genotype and airborne particulate matter could work on the same oxidative stress and inflammatory pathways, which might jointly induce the risk of cognitive decline and dementia [16]. However, existing air pollution-dementia studies have primarily examined interactions between individual air pollutants and genetic risk [7], and no studies have assessed the possible combined or interaction effects of joint air pollution exposures and genetic susceptibility on the risk of dementia.

In the study, we leveraged the comprehensive information on air pollution concentrations from the UK Department for Environment, Food and Rural Affairs (DEFRA), and used large samples from the UK Biobank [17] to assess the association between the four air pollutants (i.e., PM_2.5_, PM_10_, NO_2_, and NO_x_) and the risk of dementia. We further evaluated the association between joint exposure to these air pollutants and dementia by calculating a weighted score via a cross-validation analysis to avoid the problem of data reuse. We performed an inverse-variance weighted meta-analysis to create a pooled effect and compute a calibrated *p*-value for the combined effect with the harmonic mean *p*-value (HMP) aggregation approach. Further, we evaluated the genetic influence on dementia, the joint association between air pollution score and genetic predisposition to dementia, and the gene-air pollution interaction on dementia by including polygenic risk score (PRS) or *APOE ε4* genotype.

## Methods

### Study Design and Participants in the UK Biobank Cohort

We performed our analyses in the UK Biobank cohort [17], which was a large population-based prospective study with over half a million participants aged 40–69 years recruited in 2006–2010. Clinical, genetic, and biochemical data for each participant were obtained from various assessment centers across the UK through questionnaires, physical measurements, sample testing, genotyping, and linked electronic health reports. All included participants provided written informed consent at baseline, and ethical approval was granted by the North West Multicenter Research Ethics Committee.

**FIGURE 1:**
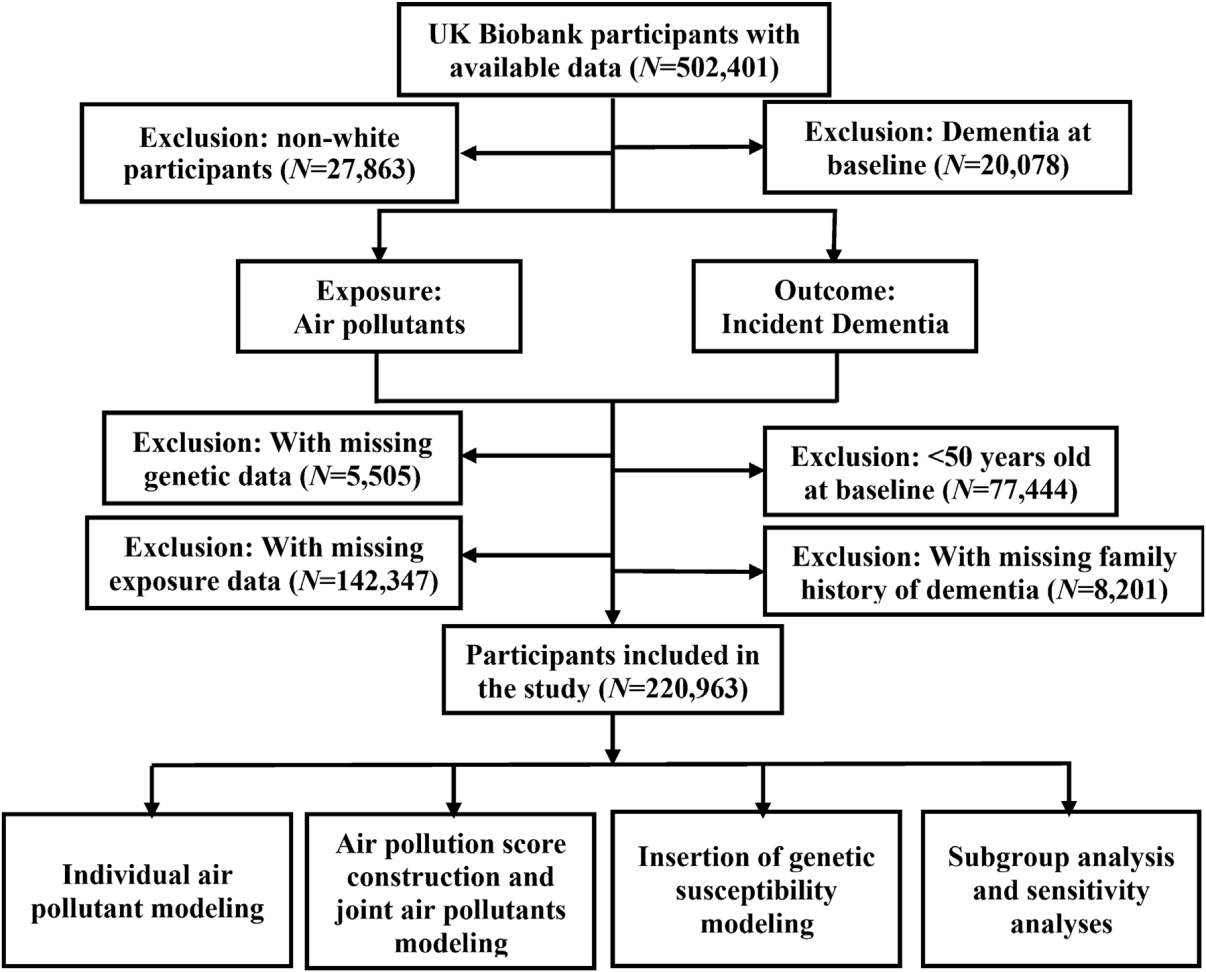
Flowchart of participant enrollment in the present study to assess the association between long-term joint exposure to multiple air pollutants and incident dementia with data available from the United Kingdom Biobank cohort (United Kingdom, 2006–2022).

We only included participants of white ethnic background, and further filtered out participants with dementia diagnosed before baseline. As dementia was more likely to occur in older adults [18], we also excluded participants aged <50 years at recruitment as done in previous work [19], leaving 220,963 participants. The flowchart illustrating the process of participant selection is shown in Figure 1.

### Exposure to Air Pollution and Concentration Measurement

We extracted high resolution air pollution data from the UK DEFRA, including PM_2.5_, PM_10_, NO_2_, and NO_x_. Air pollution levels were assessed annually at 1 × 1 km resolution under DEFRA’s Modelling of Ambient Air Quality contract. The annual concentration data of PM_10_, NO_2_, and NO_x_ from 2001 to 2021 and the concentration data of PM_2.5_ from 2002 to 2021 were collected.

Following previous studies [20, 21], we calculated the mean concentration of pollutant exposure for each participant from 3 years prior to recruitment to the incident dementia, death, or the end of follow-up, whichever came first. Instead of using baseline exposures, we here chose pollutant exposures from 3 years before the recruitment in order to more accurately represent air pollution exposure levels during a long-term follow-up.

### Ascertainment of Dementia Events and Survival Time

Diagnoses of new-onset incident dementia cases were determined via the International Classification of Diseases (ICD9 and ICD10) coding system (Supplementary Table S1). The accuracy of dementia ascertainment has been validated previously [22]. The diagnosis date was defined as the earliest reported date when the dementia event occurred. The follow-up time for each participant was calculated from the date of attendance at the assessment center to diagnosis date of a new-onset dementia event, the date of death, loss to follow-up, or end of the follow-up (19 July 2022), whichever came first.

### Covariate Selection and Missing Value Treatment

A range of sociodemographic characteristics and lifestyle factors were identified *a priori* as covariates through literature search to adjust for potential confounding influences, including age (years), sex (male or female), education (college degree or without college degree), income (<£31,000 or ≥£31,000) [23], body mass index (BMI) (kg/m^2^), drinking status (never, former, or current drinker), smoking status (never, former, or current smoker), physical activity (assessed through the international physical activity questionnaire (IPAQ) and divided into high, moderate and low levels [24]), healthy diet score (calculated based on daily dietary factors ranging from 0 to 5 [25]), urban residency (urban or rural), Townsend deprivation index (TDI) [26], family history of dementia (yes or no), and the first ten genetic principal components.

We excluded participants with missing values in air pollution, genetic data, and family history of dementia, but imputed other missing covariates by the method of multivariate imputations by chained equations via the R MICE package [27]. Finally, a total of 3,385 dementia cases and 217,578 controls were obtained for analysis.

### Genetic Susceptibility of Dementia

#### Construction of Polygenic Risk Score

We selected 29 SNPs to construct PRS (without the *APOE* genotype) for dementia (Supplementary Table S2); all these genetic variants were previously reported in large-scale GWASs of AD and dementia in individuals of European ancestry [12]. PRS was calculated as an overall measure of genetic risk of dementia: PRS = $\sum_{j=1}^{29}G_{j}{\hat{\beta }}_{j}$ , where *G*_*j*_ was the genotype representing the number of risk alleles of the *j*th SNP, and ${\hat{\beta }}_{j}$ was the effect derived from [12]. Details on genotyping and quality control in the UK Biobank have been studied previously [28]. Participants were further divided into three groups based on the quantile of PRS: low (1st quantile), moderate (2nd quantile), and high (3rd quantile).

#### APOE Genotyping

The *APOE* genotypes were determined by a combination variant of rs429358 and rs7412 [25]. Based on the number of *APOE ε4* alleles, participants can be divided into three different groups: high-risk group (*APOE ε4* dosage = 2, *ε4*/*ε4*), intermediate-risk group (*APOE ε4* dosage = 1, *ε3*/*ε4*), and low-risk group (*APOE ε4* dosage = 0, *ε2*/*ε2*, *ε2*/*ε3*, and *ε3*/*ε3*) [29].

### Statistical Analyses and Methods

#### Cox Model and Restricted Cubic Spline Regression

Baseline sociodemographic and lifestyle were summarized and stratified by dementia status (with or without incident dementia). Continuous or categorical variables in the two statuses were compared by Student′s *t* or χ^2^ test when appropriate. Cox model was employed to evaluate the associations between individual air pollutants and incident dementia, with hazard ratio (HR) and 95% confidence interval (CI) reported. The crude model (Model 1) was only adjusted for age and sex. The main model (Model 2) was further adjusted for education, BMI, drinking status, smoking status, physical activity, healthy diet score, urban residency, TDI, and family history. Schoenfeld’s residuals were used to test the proportional hazards assumption of Cox model [30], but no violations were observed.

Restricted cubic spline function with three knots was used to characterize the exposure-response curves between individual air pollutants and the risk of incident dementia. As non-linear relations were observed, the HR per interquartile range (IQR) increase or in quartile (Q1-Q4) and its 95% CI were reported.

#### Construction of Weighted Air Pollution Score Via Cross-Validation Analysis

We calculated Spearman’s correlation coefficients to assess correlation among the four air pollutants (Supplementary Table S3), and observed high collinearity. Statistically, the collinearity could lead to unreasonable effect estimates when we evaluated their joint effect on incident dementia if including them simultaneously (see below). To avoid the unwanted problem resulting from collinearity, we built a weighted air pollution score [25]. When constructing the weighted air pollution score, we noted the issue of data reuse in previous work [25, 31], which would lead to false association discovery and overestimated effect. The utilization of external data similar to the UK Biobank cohort to generate air pollutant weights is a natural way to avoid data reuse; however, such data are unavailable for us. We thus performed a cross-validation analysis to overcome this issue.

Specifically, we first randomly divided the participants into ten batches, with nine batches as the training data and the remaining one batch as the test data. Cox regression was conducted on the training data to obtain the effect (i.e., weight) of each air pollutant on incident dementia. Then, air pollution score was calculated in the test data as: ( ${\hat{\alpha }}_{\text{PM}2.5}$ × PM_2.5_ + ${\hat{\alpha }}_{\text{PM}10}$ × PM_10_+ ${\hat{\alpha }}_{\text{NO}2}$ × NO_2_ + ${\hat{\alpha }}_{\text{NO}x}$ × NO_x_) × (4/sum of these weights), where $\hat{\alpha }$ was the estimated effect of each air pollutant. This process was repeated ten times for each batch of data. Next, the association between air pollution score and incident dementia was evaluated via the crude model and the main model. Finally, the inverse-variance weighted meta-analysis was employed to produce a pooled effect, with the *p*-value obtained via the HMP method [32, 33].

#### Interaction Analysis Between Genetic Susceptibility and Air Pollution Score

The crude model and the main model were applied to estimate the joint effects of air pollution score and genetic susceptibility on incident dementia, adjusted for the first ten genetic principal components. Participants were divided into 12 groups based on quartiles of air pollution score and tertiles of PRS or *APOE ε4* dosage. The HR of incident dementia was estimated in different groups compared to those with the lowest quartile of air pollution score and low genetic risk.

We also evaluated interactions on both multiplicative and additive scales (Zhang et al. 2023a). The HR of the multiplicative term, the relative excess risk due to interaction (RERI), and the attributable proportion due to interaction (AP) were used as the measures of interaction on the multiplicative and additive scales, respectively.

#### Subgroup and Sensitivity Analyses

To identify specific participants susceptible to air pollution score, we performed a series of subgroup analyses. We repeated the analysis in sub-studies stratified by sex (male or female), age (<60 or ≥60 years old) [34], income (<£31,000 or ≥£31,000) [23], education (college degree or without college degree), TDI (<−2.4 or ≥−2.4, the average of all participants) [35], smoking status (former smoking or non-smoking), drinking status (former drinking or non-drinking), physical activity (low, moderate, or high), healthy diet score (0–2, 3–5), BMI (<25, 25∼30, or ≥30 kg/m^2^) [36], and urban residency (urban or rural).

Several sensitivity analyses were implemented to examine the robustness of our findings: (i) referring to a previous study [37], we divided participants into different genetic risk groups according to PRS quintiles: low (1st quintile), moderate (2nd-4th quintiles), and high (5th quintile); (ii) to minimize the induction time bias, we excluded participants who follow-up time were less than 2 years; (iii) we extended our study population by including participants aged <50 years at baseline who were excluded in main analysis; we also further restricted our study participants to those aged ≥60 years at baseline to explore the influence of age range [38]; (iv) considering the occurrence of several diseases and drugs use might be associated with the incident dementia, we included participants’ comorbidities history (including stroke, hypertension, atrial fibrillation and cardiovascular disease) [39], and drugs history (including anti-hypertensive drugs, statins and insulin) [40, 41] as covariates (Supplementary Tables S12–13), to examine the robustness of the association between air pollution score and the incident dementia.

## Results

### Characteristics of the Participants

The baseline characteristics of all included participants are shown in Table 1. Among 220,963 participants without any dementia events at baseline, 3,385 incident dementia cases were recorded during a median follow-up of 13.4 years (2,959,882 person-years). Compared to those without incident dementia, participants with incident dementia trended to be older, predominantly male, and have higher BMI and TDI, and lower income. In addition, they were more likely to be active smokers, but less likely to be active drinkers or to have a healthy diet and high physical activity, more live in urban, and more likely to be *APOE ε4* carriers. The mean (IQR) concentrations of PM_2.5_, PM_10_, NO_2_, and NO_x_ were 9.9 (2.3), 14.8 (3.2), 18.1 (6.9), and 26.9 (12.0) mg/m^3^, respectively.

**TABLE 1:** Baseline characteristics of participants stratified by dementia status in the United Kingdom Biobank cohort study (United Kingdom, 2006–2022).

	All participants (*N* = 220,963)	Incident dementia (*N* = 3,385)	No dementia (*N* = 217,578)	*p* Values
Age at recruitment (years)	59.9 ± 5.4	64.5 ± 4.1	59.9 ± 5.4	<0.001
Duration of follow-up (years)	13.4 ± 1.1	9.2 ± 2.8	13.5 ± 0.9	<0.001
Gender (%)				
Male	97,504 (44.1%)	1,638 (48.4%)	95,866 (44.1%)	<0.001
Female	123,459 (55.9%)	1,747 (51.6%)	121,712 (55.9%)	
BMI (mean ± SD), (kg/m^2^)	27.4 ± 4.7	27.5 ± 4.9	27.4 ± 4.7	0.284
TDI (mean ± SD)	−1.6 ± 2.9	−1.2 ± 3.2	−1.6 ± 2.9	<0.001
Qualifications (%)				
With college	69,169 (31.3%)	721 (21.3%)	68,488 (31.5%)	<0.001
Without college	106,473 (48.2%)	1,447 (42.7%)	105,026 (48.3%)	
Income (%)				
Less than £31,000	95,791 (43.4%)	1,982 (58.6%)	93,809 (43.1%)	<0.001
Equal to or above £31,000	93,148 (42.2%)	648 (19.1%)	92,500 (42.5%)	
Smoking status (%)				
Never	117,126 (53.0%)	1,612 (47.6%)	115,514 (53.1%)	<0.001
Former	83,153 (37.6%)	1,420 (41.9%)	81,733 (37.6%)	
Current	19,878 (9.0%)	326 (9.6%)	19,552 (9.0%)	
Drinking status (%)				
Never	7,481 (3.4%)	211 (6.2%)	7,270 (3.3%)	<0.001
Former	7,560 (3.4%)	215 (6.4%)	7,345 (3.4%)	
Current	205,749 (93.1%)	2,952 (87.2%)	202,797 (93.2%)	
Physical activity (%)				
Low	32,614 (14.8%)	490 (14.5%)	32,124 (14.8%)	0.275
Moderate	73,528 (33.3%)	1,070 (31.6%)	72,458 (33.3%)	
High	70,901 (32.1%)	981 (29.0%)	69,920 (32.1%)	
Healthy diet score (%)				
0	3,061 (1.4%)	48 (1.4%)	3,013 (1.4%)	0.864
1	14,856 (6.7%)	232 (6.9%)	16,624 (7.6%)	
2	35,211 (15.9%)	526 (15.5%)	34,685 (15.95)	
3	51,897 (23.5%)	769 (22.7%)	51,128 (23.5%)	
4	52,794 (23.9%)	754 (22.3%)	52,040 (23.9%)	
5	31,390 (14.2%)	458 (13.5%)	30,932 (14.2%)	
Family history of dementia (%)				
yes	30,171 (13.7%)	461 (13.6%)	29,710 (13.7%)	0.952
no	190,792 (86.3%)	2,924 (86.4%)	187,868 (86.3%)	
*APOE ε4* dosage (%)				
0	158,674 (71.8%)	1,532 (45.3%)	157,142 (72.2%)	<0.001
1	51,699 (23.4%)	1,390 (41.1%)	50,309 (23.1%)	
2	5,085 (2.3%)	388 (11.5%)	4,697 (2.2%)	
Urban residency				
urban	185,658 (84.0%)	2,937 (86.8%)	182,720 (84.0%)	<0.001
rural	33,571 (15.2%)	430 (12.7%)	331,41 (15.2%)	

Note: Continues variables displayed as means ± SD, and categorical variables are displayed as numbers (percentages). BMI, body mass index; TDI, townsend deprivation index; APOE, apolipoprotein E. The missing data were not reported here. Continuous or categorical variables in different groups were compared by Student′s *t* or χ^2^ test when appropriate.

### Association Between Individual Air Pollutants and Dementia

**FIGURE 2:**
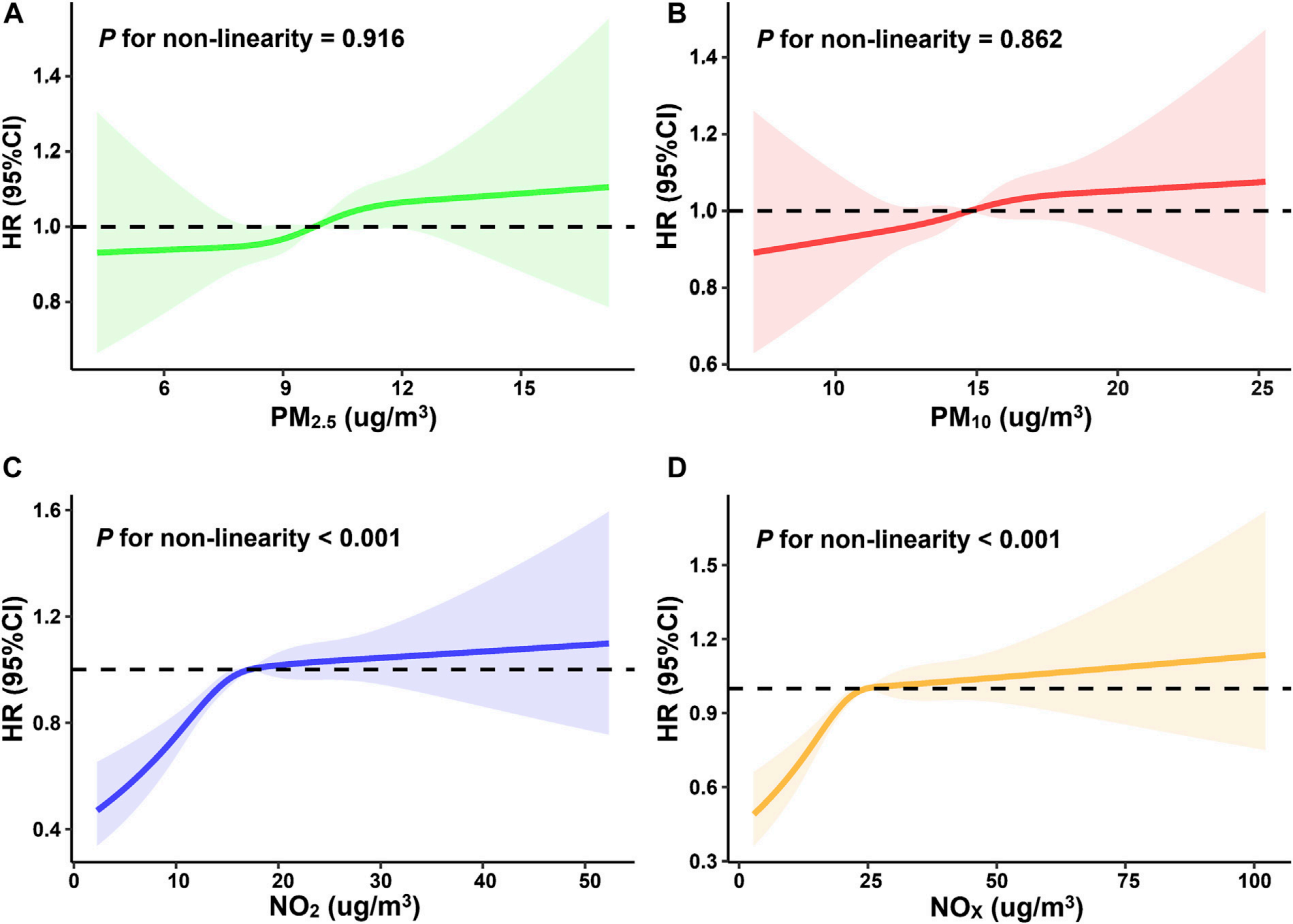
Restricted cubic spline curves for the influence of four individual air pollutants (**(A)**: PM_2.5_, **(B)**: PM_10_, **(C)**: NO_2_, **(D)**: NO_X_) on incident dementia (United Kingdom, 2006–2022).

The restricted cubic spline curves indicated that the associations between NO_2_, NOx and incident dementia were non-linear (*P*_nonlinearity_<0.001) (Figure 2). We observed that PM_2.5_, PM_10_, NO_2_, and NO_x_ were all associated with increased risk of occurring dementia (Table 2). In terms of the main model, per IQR increase of PM_2.5_, PM_10_, NO_2_, NO_x_ would result in 6% (95%CI) (2%∼12%), 5% (1%∼10%), 10% (5%∼14%), and 6% (4%∼13%) higher risk of dementia, respectively. Compared to Q1 (the lowest air pollution), the Q4 (the highest air pollution) HRs (95%CI) of incident dementia for the four air pollutants were 1.17 (1.06∼1.29), 1.13 (1.02∼1.24), 1.20 (1.08∼1.33), and 1.20 (1.08∼1.34), respectively.

**TABLE 2:** Adjusted hazard ratio and 95% confidence interval for individual air pollution concentrations with the risk of incident dementia (United Kingdom, 2006–2022).

	Air pollutant	per IQR increase^a^	Air pollution concentrations (quintiles)				*p* For trend
			Q1^b^	Q2^b^	Q3^b^	Q4^b^	
Model 1	PM_2.5_	1.07 (1.02∼1.12)	1.00	1.07 (0.97∼1.18)	1.09 (0.99∼1.20)	1.19 (1.08∼1.31)	3.45 × 10^−4^
	PM_10_	1.06 (1.01∼1.11)	1.00	1.07 (0.97∼1.18)	1.08 (0.98∼1.18)	1.14 (1.04∼1.26)	8.80 × 10^−3^
	NO_2_	1.12 (1.08∼1.16)	1.00	1.17 (1.06∼1.29)	1.29 (1.17∼1.53)	1.30 (1.18∼1.43)	3.71×10^−8^
	NO_x_	1.11 (1.07∼1.15)	1.00	1.18 (1.07∼1.30)	1.27 (1.15∼1.40)	1.30 (1.18∼1.44)	1.10 × 10^−7^
Model 2	PM_2.5_	1.06 (1.02∼1.11)	1.00	1.04 (0.95∼1.15)	1.07 (0.97∼1.18)	1.17 (1.06∼1.29)	1.30 × 10^−3^
	PM_10_	1.05 (1.01∼1.10)	1.00	1.04 (0.95∼1.15)	1.06 (0.96∼1.16)	1.13 (1.02∼1.24)	0.017
	NO_2_	1.10 (1.05∼1.14)	1.00	1.09 (0.98∼1.21)	1.19 (1.07∼1.32)	1.20 (1.08∼1.33)	5.90 × 10^−4^
	NO_x_	1.09 (1.02∼1.13)	1.00	1.11 (1.00∼1.23)	1.16 (1.05∼1.29)	1.20 (1.08∼1.34)	1.01 × 10^−3^

Note:

^a^ Each air pollution concentration was treated as continuous variable and the HR, per interquartile range (IQR) increase and its 95%CI, were reported.

^b^ Each air pollution concentration was divided into four categories (Q1-Q4), and HR, per one quartile increment and its 95%CI, were reported. Q1: the first quartile; Q2: the second quartile; Q3: the third quartile; Q4: the least quartile.

Model 1: adjusted for age and sex.

Model 2: included covariates in model 1 and additionally adjusted for education agree, BMI, drinking status, smoking status, physical activity, healthy diet score, urban residency, TDI, and family history.

### Association Between Air Pollution Score and Dementia

The Spearman’s correlations among the four air pollutants are shown in Supplementary Table S3, which indicated strong dependence among them. Due to this collinearity of air pollutants, we found that introducing them into the model simultaneously leaded to unreasonable effect estimates (both increased and reduced risk associations existed) when evaluating their joint influences (Supplementary Table S4). Therefore, it was necessary to create an air pollution score for assessing the combined effect of these air pollutants on dementia.

The restricted cubic spline curves for the association of air pollution score with the risk of incident dementia are shown in Supplementary Figure S1, the Kaplan-Meier curves are displayed in Supplementary Figure S2, with significant differences in the cumulative risk of dementia according to different quartiles of air pollution score (*P*_Log-rank_ < 0.0001). The associations between air pollution score and incident dementia are given in Table 3. In the main model, the HR of incident dementia per IQR increase in air pollution score was 1.13 (95%CI 1.07∼1.18). In comparison to those with Q1 (the lowest joint air pollution exposure), participants with Q4 (the highest joint air pollution exposure) had a HR of 1.26 (95%CI 1.13∼1.40). Further, we observed that the effect of air pollution score was higher rather the impacts of individual air pollutants, with an average of 6.0% or 5.5% greater risk in terms of model 1 or model 2, respectively.

**TABLE 3:** Adjusted hazard ratio and 95% confidence interval for air pollution score with the risk of incident dementia (United Kingdom, 2006–2022).

	per IQR increase^a^	Air pollution concentrations (quintiles)				*p* For trend
		Q1^b^	Q2^b^	Q3^b^	Q4^b^	
Model 1	1.15 (1.11∼1.20)	1.00	1.19 (1.08∼1.31)	1.27 (1.15∼1.40)	1.34 (1.21∼1.47)	8.73 × 10^−10^
Model 2	1.13 (1.07∼1.18)	1.00	1.12 (1.00∼1.24)	1.19 (1.07∼1.33)	1.26 (1.13∼1.40)	2.17 × 10^−5^

Note:

^a^ Each air pollution concentration was treated as continuous variable and the HR, per interquartile range (IQR) increase and its 95%CI, were reported.

^b^ Each air pollution concentration was divided into four categories (Q1-Q4), and HR, per one quartile increment and its 95%CI, were reported. Q1: the first quartile; Q2: the second quartile; Q3: the third quartile; Q4: the least quartile.

Model 1: adjusted for age and sex.

Model 2: included covariates in model 1 and adjusted for additionally education agree, BMI, drinking status, smoking status, physical activity, healthy diet score, urban residency, TDI, and family history.

### Influence of Genetic Susceptibility on Dementia

We detected a significant association of genetic susceptibility with the risk of incident dementia after controlling potential covariates. The restricted cubic spline curves for the association of PRS with the risk of incident dementia are shown in Supplementary Figure S3. On average, the PRS was associated with approximately 25% (95%CI 21%∼28%) higher risk of incident dementia, and the participants with high-risk group (*APOE ε4* dosage = 2) had greater risk of incident dementia, with a HR of 8.64 (95%CI 7.73∼9.67).

**FIGURE 3:**
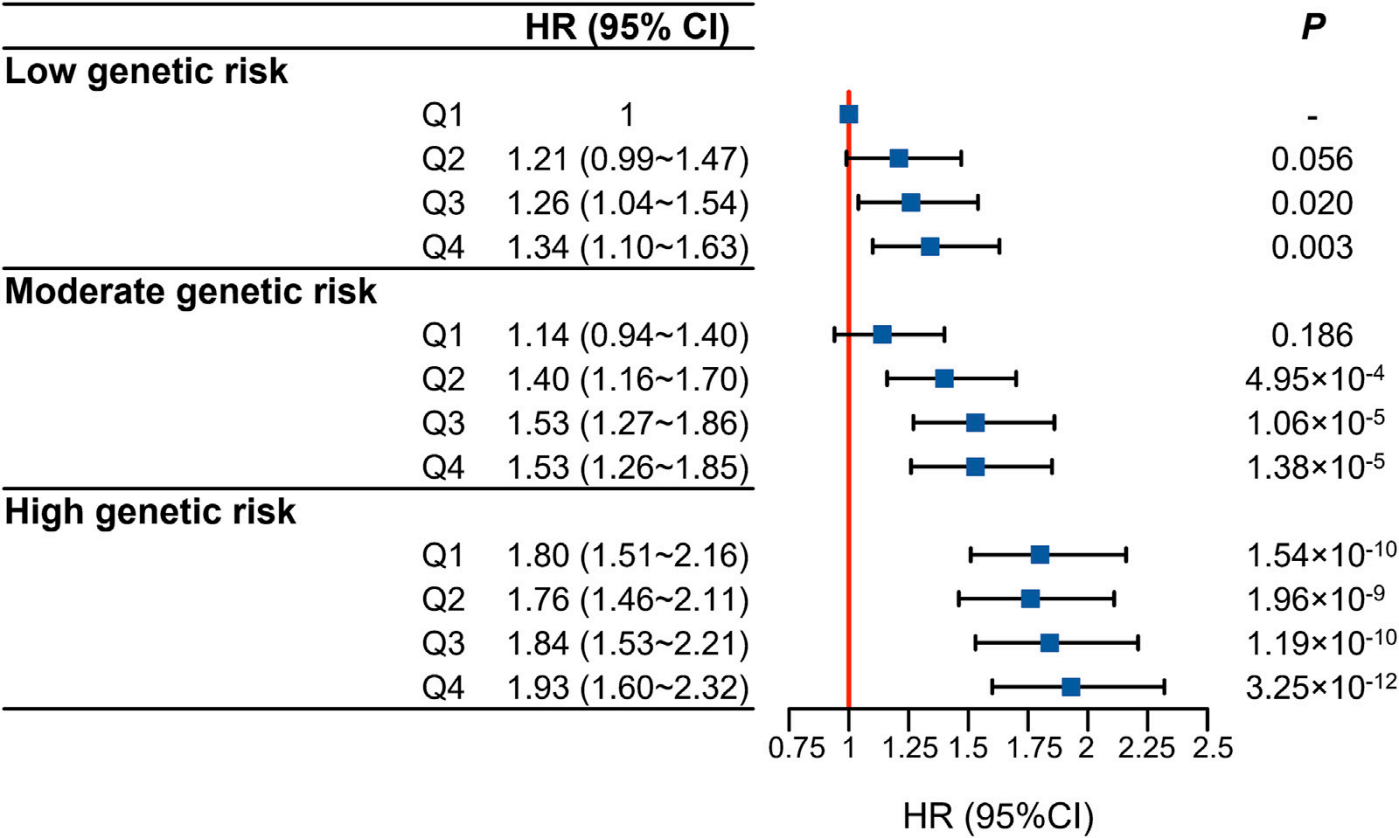
Joint association of air pollution score (in quartiles) and dementia polygenic risk score (in tertiles) with the risk of incident dementia (United Kingdom, 2006–2022).

We further evaluated the joint association of air pollution score and genetic predisposition with incident dementia. The results showed that, compared to those with the lowest PRS and lowest air pollution score, participants with the highest PRS and air pollution score had the greatest risk of incident dementia (HR = 1.93, 95%CI 1.60∼2.32) (Figure 3). Similar association patterns were also seen in participants with *APOE ε4* dosage. Compared to those with low-risk group (*APOE ε4* dosage = 0) and the lowest air pollution score, participants with high-risk group (*APOE ε4* dosage = 2) and the highest air pollution score had the greatest risk of incident dementia (HR = 10.5, 95%CI 9.31∼13.1) (Supplementary Table S5). Unfortunately, when examining whether there were interactions between air pollution score and genetic predisposition to incident dementia, we did not detect statistically significant multiplicative (*p* = 0.193) or additive interaction between air pollution score and genetic susceptibility to dementia (Supplementary Table S6).

### Results of Subgroup and Sensitivity Analyses

In the subgroup analyses conducted at different levels of a given covariate, air pollution score remained robustly associated with the risk of occurring incident dementia. In particular, we found that the association between air pollution score and incident dementia posed stronger among female participants, or those with low years old (<60 years), rural, high income (≥£31,000 annually), never-smoker, drinker, living in high deprived areas (TDI ≥−2.4), high BMI (≥30), and low physical activities (Supplementary Table S7). Furthermore, compared to Q1 (the lowest air pollution), the Q4 (the highest air pollution) HR of incident dementia for air pollution score was the highest in all subgroups of the population, with the exception of never drinker, low BMI (<25), high education degree (college degree or above), high healthy diet score (3–5), living in high deprived areas (TDI ≥−2.4), and low physical activities.

In the sensitivity analyses, similar association patterns were discovered when different PRS cut-offs were applied to divide participants into distinct groups as no interactions were identified between air pollution score and genetics (Supplementary Table S8). Similar relations were seen when participants with less than 2 years of follow-up were excluded (Supplementary Table S9). Changes to study population selection by extending the age range to <50 years or further restricting it to ≥60 years did not result in substantial changes in effect and association (Supplementary Tables S10–11). After considering participants’ comorbidities history and drugs history, the association between air pollution score and the incident dementia remained robust (Supplementary Table S14).

## Discussion

### Summary of Our Study

In this prospective cohort study using the UK Biobank [17], we have evaluated the association of four major air pollutants (i.e., PM_2.5_, PM_10_, NO_2_, and NO_x_) with the incident dementia, and evaluated joint exposure to them through creating an air pollution score. We revealed that air pollution score was strongly associated with increased risk of dementia events, and that this adverse effect could be reinforced by genetic susceptibility to dementia.

### Comparison to Previous Studies

#### Comparison With Previous Studies on Individual Air Pollutants

Epidemiological research has widely investigated the health effects of long-term air pollution exposure on dementia [8]. In particular, PM_2.5_ is the most frequently studied air pollutant, and has been reported a positive association with dementia [8, 9, 42]; this finding is consistent with ours. However, a conclusive correlation between air pollution exposure and dementia remains elusive since several cohort studies reported null relations between PM_2.5_ and the incidence of dementia [10, 43].

For PM_10_, one Italian study explored its influence on dementia but failed to detect a significant relation [10]. In addition, gaseous air pollutants (e.g., NO_2_) were evaluated previously [44]; one study showed the strongest association between NO_2_ and incident dementia [9], but another study did not repeat such an association. Similarly, the relation between NO_x_ and dementia was also undefined [8].

Overall, the current epidemiological evidence regarding the association between air pollution exposure and dementia, regardless of the type of dementia, remains sparse and inconsistent [38]. In contrast to previous studies, our work explicitly indicated the substantial connection between these air pollutants and the risk of incident dementia. Moreover, prior studies only focused on the effects of individual air pollutants but ignored the combined effects of multiple pollutant exposures on dementia; whereas we investigated the joint influence of various air pollutants.

#### Comparison With Existing Studies on Joint Exposure to Air Pollutants

The importance of evaluating the health effects of multi-pollutant exposures has received increasing attention in recent years [45]. A lot of studies have found that air pollution score reflects a more comprehensive measure of combined exposure to diverse air pollutants than any individual air pollutants [25, 46]. Existing studies have proposed statistical methods similar to the approach used here to estimate the combined effects of multiple pollutant exposures on human health by summing air pollutant concentrations in a weighted manner. For example, Wang *et al.* identified that air pollution score was associated with greater risk of incident dementia in a dose-response fashion [25], independent of traditional risk factors; Gao *et al.* uncovered that the air pollution index reflecting joint effects of pollutants was positively related to the risk of mental disorders [46]. Similar algorithms were also applied to assess joint exposure to other environmental risk factors and dietary factors [47]. Further, such scoring algorithms make epidemiological findings easier to be interpreted and promote public health preventive measures.

#### Current Findings in the Present Study

Currently, dementia was incurable and its etiology remained unclear; thus, early prevention is critical. Potentially biological mechanisms indicate that exposure to air pollution may have deleterious effects on the central nervous system through the induction of oxidative stress and neuroinflammation, potentially leading to cognitive impairment and the development of dementia [48]. As the connection between air pollution score and dementia risk is much stronger than that between individual air pollutants, we assume that various air pollutants likely have a superimposed effect on dementia risk through similar biological mechanisms.

We also assessed the joint association of air pollution and genetic susceptibility with the risk of dementia events and discovered supportive evidence for a modification role of genetic risk in the air pollution-dementia association. Currently, only a few studies have investigated whether the association between air pollution exposure and dementia could be regulated by genetic factors, particularly by *APOE* status, and none have reported a significant interaction [49]. In our study, we considered both *APOE* and non-*APOE* alleles and calculated PRS as a combined measure of genetic predisposition to dementia. We observed that, although the interaction between air pollution score and genetic susceptibility was insignificant, the higher air pollution score, the higher the genetic risk and the increased risk of dementia; consistent results were observed when using *APOE* genotype.

### Public Health Implications

Our findings have significant public health implications. First, our study supported that exposure to air pollution, especially pollutants PM_2.5_, PM_10_, NO_2_, and NO_x_, leaded to increased risk of dementia, suggesting that exposure to these pollutants needed to be avoided as much as possible, which could help reduce the personal financial and social burdens of dementia.

Second, we revealed that multiple air pollutants were associated with greater risk of dementia than exposure to individual air pollutants, indicating a potential additive or synergistic effect of air pollutants on incident dementia [50], which prompts us to give more consideration in future air quality standards/guidelines or policy development not only to individual air pollutants, but also to the relevance of mixtures of air pollutants to the same emission sources.

Third, our results implied that participants with higher genetic risk of dementia tended to have higher risk of dementia induced by air pollution. Therefore, people with high genetic susceptibility to dementia should be more concerned about the hazard of air pollution.

### Strengths of this Study

The present study has several advantages. First, our study was a prospective study that assessed the association between joint exposure to ambient air pollutants and the risk of incident dementia. The new findings on association between joint air pollutants and dementia would lead to the development of new prevention strategies by considering multiple air pollutants together.

Second, our study had a relatively long follow-up time (mean 13.4 years) and a sufficiently large sample size. In addition, our analysis considered important individual-level confounders (e.g., smoking status and TDI) and fully accounted for genetic susceptibility factors (including *APOE* and non-*APOE* loci), which allowed us to prospectively estimate the risk of dementia and modifications of genetic factors.

Finally, in contrast to previous studies constructing air pollution score and assessing the association between air pollution score and health outcomes in the same whole data [25, 31], which would result in the typical issue of data reuse such as inflated type I error and overestimated effect. We instead performed a cross-validation analysis to obtain effect estimates and *p*-values, and then used the inverse-variance weighted meta-analyses and HMP to obtain the pooled effect and *p*-value, respectively, avoiding the problem of data reuse mentioned above [51]. The advantage of HMP is that it effectively aggregates a group of correlated *p*-values based on the model-averaged mean maximum likelihood ratio and is robust against unknown positive dependence [32, 33].

### Limitations of the Present Work

The present study also has several potential limitations. First, all the UK Biobank participants were recruited voluntarily and participants were healthier than the general population [52], which may underestimate the adverse effects of air pollutions.

Second, only four major air pollutants were included in our study, while the effects of other major air pollutants such as ozone and sulfur dioxide on dementia need to be considered for study in future studies [53].

Third, we only constructed the PRS for whites; it is necessary to further determine whether these results can be generalized to other populations [54]. Finally, air pollution exposure was only estimated at the baseline address of the participants and future studies should take into account the mobility of the dwelling [35, 42].

### Conclusion

Our study provides evidence that joint exposure to multiple air pollutants substantially increases the risk of dementia, especially among individuals with high genetic susceptibility.

## Abbreviation

APOE, apolipoprotein *E* gene; PRS, polygenic risk score; CI, confidence interval; HR, hazard ratio; GWAS, genome-wide association study; SD, standard deviation; BMI, body mass index; TDI, Townsend deprivation index; SNP, single nucleotide polymorphism; HMP, harmonic mean *p*-value; AD, Alzheimer’s disease.
