# Supplementary material for: Joint Exposure to Multiple Air Pollutants, Genetic Susceptibility, and Incident Dementia: A Prospective Analysis in the UK Biobank Cohort
Source: Int J Public Health. 2024 Feb 15;69:1606868. doi: 10.3389/ijph.2024.1606868 (PMC10901982; doi:10.3389/ijph.2024.1606868)
Supplement: Supplementary file 1 [file DataSheet1.docx]

**Supplementary File**

# Table S1. Definitions and sources of information for dementia in the United Kingdom Biobank (United Kingdom, 2006-2022).

| Dementia | ICD-9 | ICD-10 |
| --- | --- | --- |
| Alzheimer's disease | 331.0 | F00, F00.0, F00.1, F00.2, F00.9, G30, G30.0, G30.1, G30.8, G30.9 |
| Vascular dementia | 290.4 | F01, F01.0, F01.1, F01.2, F01.3, F01.8, F01.9, I67.3 |
| Other codes for all-cause dementia | 290.2, 290.3, 291.2, 294.1, 331.1, 331.2, 331.5 | A81.0, F02, F02.0, F02.1, F02.2, F02.3, F02.4, F02.8, F03, F05.1, F10.6, G31.0, G31.1, G31.8 |

Note: ICD: International Classification of Disease.

# Table S2. Summary of the 29 genomic loci associated with dementia (United Kingdom, 2006-2022).

| No | Chr | SNP | Position | Major Alleles | Minor Alleles | Beta | P |
| --- | --- | --- | --- | --- | --- | --- | --- |
| 1 | 1 | rs4575098 | 161155392 | A | G | 0.016412 | 1.9E-10 |
| 2 | 1 | rs6656401 | 207786828 | A | G | 0.025014 | 2.58E-18 |
| 3 | 2 | rs4663105 | 127891427 | C | A | 0.031095 | 1.45E-44 |
| 4 | 2 | rs10933431 | 233981912 | G | C | -0.01544 | 7.62E-10 |
| 5 | 4 | rs6448453 | 11026028 | A | G | 0.014705 | 1.98E-09 |
| 6 | 4 | rs7657553 | 11723235 | A | G | 0.00484 | 0.044664 |
| 7 | 6 | rs9269853 | 32550322 | A | C | 0.013069 | 3.04E-08 |
| 8 | 6 | rs9381563 | 47432637 | C | T | 0.014451 | 1.99E-10 |
| 9 | 7 | rs1859788 | 99971834 | A | G | -0.0184 | 1.8E-15 |
| 10 | 7 | rs11763230 | 1.43E+08 | T | C | -0.01577 | 5.37E-09 |
| 11 | 8 | rs4236673 | 27464929 | A | G | -0.02016 | 1.48E-19 |
| 12 | 10 | rs11257242 | 11721119 | C | G | -0.01001 | 8.11E-06 |
| 13 | 11 | rs7935829 | 59942815 | G | A | -0.0176 | 2.7E-15 |
| 14 | 11 | rs10792832 | 85867875 | A | G | -0.01948 | 4.5E-18 |
| 15 | 11 | rs11218343 | 1.21E+08 | C | T | -0.03593 | 8.12E-12 |
| 16 | 14 | rs12590654 | 92938855 | A | G | -0.01483 | 1.32E-10 |
| 17 | 15 | rs442495 | 59022615 | C | T | -0.01372 | 1.22E-09 |
| 18 | 15 | rs117618017 | 63569902 | T | C | 0.018027 | 3.44E-08 |
| 19 | 16 | rs59735493 | 31133100 | A | G | -0.01299 | 3.73E-08 |
| 20 | 17 | rs113260531 | 5138980 | A | G | 0.019986 | 7.91E-10 |
| 21 | 17 | rs28394864 | 47450775 | A | G | 0.012302 | 1.68E-08 |
| 22 | 17 | rs2632516 | 56409089 | C | G | -0.01078 | 7.63E-07 |
| 23 | 18 | rs8093731 | 29088958 | T | C | -0.01774 | 0.026745 |
| 24 | 18 | rs76726049 | 56189459 | C | T | 0.056797 | 3.38E-08 |
| 25 | 19 | rs4147929 | 1063443 | A | G | 0.014413 | 4.43E-07 |
| 26 | 19 | rs41289512 | 45351516 | G | C | 0.206303 | 1.5E-278 |
| 27 | 19 | rs76320948 | 46241841 | T | C | 0.034933 | 4.08E-08 |
| 28 | 19 | rs3865444 | 51727962 | A | C | -0.01376 | 5.15E-09 |
| 29 | 20 | rs6014724 | 54998544 | G | A | -0.02289 | 5.38E-10 |

Note: Chr: chromosome; SNP: single nucleotide polymorphism.

# Table S3. Spearman′s correlation coefficients among the four air pollutants in the United Kingdom Biobank (United Kingdom, 2006-2022).

| Variables | PM_2.5_ | PM_10_ | PM_2.5-10_ | NO_2_ |
| --- | --- | --- | --- | --- |
| PM_2.5_ | 1.00 | - | - | - |
| PM_10_ | 0.99^*^ | 1.00 | - | - |
| NO_2_ | 0.78^*^ | 0.78^*^ | 1.00 | - |
| NO_x_ | 0.78^*^ | 0.78^*^ | 0.99^*^ | 1.00 |

Note: PM_2.5_: particular matter with aerodynamic diameter ≤2.5 µm; PM_10_: particular matter with an aerodynamic diameter ≤10 µm; NO_2_: nitrogen dioxide; NO_x_: nitrogen oxides. ^*^ *P*<0.001.

# Table S4. Adjusted hazard ratio and 95% confidence interval for the four air pollutants associated with dementia while introducing them into the model jointly (United Kingdom, 2006-2022).

| Air pollutants | HR (95%CI) | *P* value |
| --- | --- | --- |
| PM_2.5_ | 1.26 (1.04~1.52) | 0.021 |
| PM_10_ | 0.83 (0.73~0.96) | 0.010 |
| NO_2_ | 1.03 (0.94~1.13) | 0.524 |
| NO_X_ | 0.99 (0.94~1.05) | 0.781 |

Note: HR: hazard ratio; CI: confidence interval. This model adjusted for age, sex, education, income, BMI, drinking status, smoking status, physical activity, healthy diet score, and TDI.

# Table S5. Adjusted hazard ratio and 95% confidence interval for the joint effects of APOE and air pollution score on dementia (United Kingdom, 2006-2022).

| *APOE ε4* dosage | Quartiles | HR (95%CI) | *P* |
| --- | --- | --- | --- |
| 0 (low-risk group) |  |  |  |
|  | Q1 | 1.00 | - |
|  | Q2 | 1.16 (1.00~1.35) | 0.050 |
|  | Q3 | 1.30 (1.12~1.50) | 5.04×10^-4^ |
|  | Q4 | 1.29 (1.12~1.50) | 6.37×10^-4^ |
| 1 (intermediate-risk group) |  |  |  |
|  | Q1 | 3.06 (2.61~3.58) | 1.80×10^-43^ |
|  | Q2 | 3.35 (2.87~3.90) | 3.47×10^-54^ |
|  | Q3 | 3.48 (2.99~4.05) | 1.47×10^-57^ |
|  | Q4 | 3.87 (3.32~4.50) | 9.60×10^-69^ |
| 2 (high-risk group) |  |  |  |
|  | Q1 | 9.80 (7.76~12.38) | 1.04×10^-81^ |
|  | Q2 | 10.59 (8.49~13.21) | 2.51×10^-97^ |
|  | Q3 | 10.27 (8.15~12.94) | 7.95×10^-87^ |
|  | Q4 | 10.45 (8.31~13.14) | 1.14×10^-87^ |

Table S6. Additive interactions between air pollution score and polygenic risk score on the risk of incident dementia (United Kingdom, 2006-2022).

| Air pollution score | Moderate genetic risk | |  | High genetic risk | |
| --- | --- | --- | --- | --- | --- |
|  | RERI (95%CI) | AP (95%CI) |  | RERI (95%CI) | AP (95%CI) |
| Q2 | 0.05 (-0.26~0.36) | 0.04 (-0.18~0.25) |  | -0.26 (-0.62~0.10) | -0.15 (-0.35~0.06) |
| Q3 | 0.13 (-0.18~0.44) | 0.08 (-0.12~0.29) |  | -0.23 (-0.59~0.13) | -0.13 (-0.32~0.07) |
| Q4 | 0.05 (-0.27~0.36) | 0.03 (-0.18~0.24) |  | -0.21 (-0.58~0.15) | -0.11 (-0.30~0.08) |

Note: RERI: relative excess risk due to interaction; AP: attributable proportion due to interaction; CI: confidence interval; Q2: the second quartile; Q3: the third quartile; Q4: the fourth quartile. The bold *P*-value indicates it is significant at the level of 0.05.

Table S7. Subgroup analysis for the association of air pollution score with incident dementia (United Kingdom, 2006-2022).

| Covariates | per IQR increase ^a^ | Air pollution concentrations (quintiles) | | | | *P* for trend |
| --- | --- | --- | --- | --- | --- | --- |
|  |  | Q1^b^ | Q2^b^ | Q3^b^ | Q4^b^ |  |
| Sex |  |  |  |  |  |  |
| female | 1.14 (1.07~1.23) | 1.00 | 1.06 (0.92~1.23) | 1.13 (0.97~1.32) | 1.22 (1.05~1.42) | 7.32×10^-3^ |
| male | 1.11 (1.03~1.19) | 1.00 | 1.16 (0.99~1.35) | 1.26 (1.078 1.47) | 1.29 (1.10~1.50) | 8.78×10^-3^ |
| Age |  |  |  |  |  |  |
| <60 | 1.30 (1.12~1.52) | 1.00 | 1.02 (0.74~1.41) | 1.23 (0.90~1.69) | 1.40 (1.02~1.91) | 0.022 |
| ≥60 | 1.11 (1.05~1.17) | 1.00 | 1.12 (1.00~1.25) | 1.20 (1.07~1.35) | 1.24 (1.10~1.39) | 1.32×10^-4^ |
| Urban residency |  | |  |  |  |  |
| urban | 1.10 (1.05~1.15) | 1.00 | 0.95 (0.72~1.26) | 1.04 (0.79~1.37) | 1.30 (1.01~1.69) | 0.038 |
| rural | 1.15 (1.01~1.31) | 1.00 | 1.11 (1.00~1.24) | 1.16 (1.05~1.29) | 1.23 (1.11~1.36) | 6.51×10^-5^ |
| Income |  |  |  |  |  |  |
| <£31000 | 1.12 (1.06~1.19) | 1.00 | 1.11 (0.98~1.26) | 1.21 (1.06~1.37) | 1.28 (1.12~1.45) | 8.17×10^-5^ |
| ≥£31000 | 1.13 (1.03~1.24) | 1.00 | 1.20 (0.99~1.46) | 1.22 (0.99~1.50) | 1.24 (1.01~1.53) | 0.036 |
| Education |  |  |  |  |  |  |
| high school or below | 1.13 (1.06~1.20) | 1.00 | 1.03 (0.91~1.18) | 1.14 (1.00~1.30) | 1.25 (1.10~1.42) | 3.24×10^-4^ |
| college degree or above | 1.13 (1.03~1.23) | 1.00 | 1.27 (1.05~1.55) | 1.36 (1.12~1.66) | 1.33 (1.09~1.63) | 2.83×10^-3^ |
| Smoking |  |  |  |  |  |  |
| No | 1.14 (1.06~1.23) | 1.00 | 1.01 (0.86~1.17) | 1.14 (0.97~1.33) | 1.25 (1.07~1.46) | 0.002 |
| Yes | 1.11 (1.04~1.19) | 1.00 | 1.28 (1.10~1.48) | 1.28 (1.10~1.49) | 1.32 (1.13~1.54) | 4.01×10^-4^ |
| Drinking |  |  |  |  |  |  |
| No | 1.05 (0.88~1.26) | 1.00 | 0.85 (0.57~1.27) | 0.97 (0.65~1.45) | 0.86 (0.57~1.31) | 0.596 |
| Yes | 1.13 (1.08~1.19) | 1.00 | 1.12 (1.00~1.25) | 1.21 (1.08~1.35) | 1.28 (1.14~1.43) | 7.41×10^-6^ |
| Physical activity |  |  |  |  |  |  |
| low | 1.16 (1.04~1.30) | 1.00 | 1.26 (0.99~1.60) | 1.35 (1.05~1.72) | 1.20 (0.93~1.55) | 0.095 |
| moderate | 1.12 (1.04~1.21) | 1.00 | 1.16 (0.98~1.36) | 1.22 (1.03~1.45) | 1.32 (1.11~1.55) | 1.09×10^-3^ |
| high | 1.11 (1.03~1.20) | 1.00 | 1.00 (0.84~1.19) | 1.14 (0.96~1.36) | 1.21 (1.01~1.43) | 0.020 |
| Healthy diet score | |  |  |  |  |  |
| 0-2 | 1.13 (1.03~1.24) | 1.00 | 1.07 (0.877~1.30) | 1.10 (0.90~1.34) | 1.28 (1.05~1.56) | 0.018 |
| 3-5 | 1.13 (1.06~1.20) | 1.00 | 1.16 (1.021~1.32) | 1.27 (1.12~1.45) | 1.25 (1.09~1.42) | 2.48×10^-4^ |
| BMI |  |  |  |  |  |  |
| <25 | 1.06 (0.97~1.15) | 1.00 | 1.12 (0.90~1.36) | 1.15 (0.95~1.39) | 1.13 (0.93~1.37) | 0.187 |
| 25-30 | 1.13 (1.07~1.18) | 1.00 | 1.11 (1.00~1.24) | 1.20 (1.08~1.34) | 1.26 (1.13~1.40) | 1.57×10^-5^ |
| ≥30 | 1.15 (1.04~1.27) | 1.00 | 1.13 (0.91~1.40) | 1.24 (1.00~1.54) | 1.29 (1.03~1.60) | 0.016 |
| TDI |  |  |  |  |  |  |
| <-2.4 | 1.09 (1.01~1.18) | 1.00 | 1.05 (0.90~1.22) | 1.07 (0.91~1.26) | 1.20 (1.03~1.41) | 0.028 |
| ≥-2.4 | 1.13 (1.06~1.20) | 1.00 | 1.27 (1.10~1.46) | 1.17 (1.03~1.37) | 1.26 (1.09~1.45) | 0.003 |

# Table S8. Adjusted hazard ratio and 95% confidence interval for the joint effects of PRS (in quintiles) and air pollution score on dementia (United Kingdom, 2006-2022).

| Genetic risk groups | Quartiles | HR (95%CI) | *P* |
| --- | --- | --- | --- |
| Low (1st quintile) |  |  |  |
|  | Q1 | 1.00 | - |
|  | Q2 | 1.09 (0.84~1.41) | 0.538 |
|  | Q3 | 1.14 (0.88~1.48) | 0.305 |
|  | Q4 | 1.17 (0.90~1.51) | 0.237 |
| Moderate (2nd-4th quintiles) |  |  |  |
|  | Q1 | 1.32 (1.06~1.63) | 0.012 |
|  | Q2 | 1.51 (1.22~1.86) | 1.24×10^-4^ |
|  | Q3 | 1.50 (1.22~1.85) | 1.63×10^-4^ |
|  | Q4 | 1.63 (1.32~2.02) | 4.66×10^-6^ |
| High (5th quintile) |  |  |  |
|  | Q1 | 1.72 (1.36~2.19) | 7.58×10^-6^ |
|  | Q2 | 1.92 (1.52~2.43) | 4.15×10^-8^ |
|  | Q3 | 2.32 (1.85~2.91) | 3.55×10^-13^ |
|  | Q4 | 2.17 (1.73~2.74) | 4.37×10^-11^ |

# Table S9. Adjusted hazard ratio and 95% confidence interval for air pollution score with the risk of incident dementia among participants with follow-up time more than two years (United Kingdom, 2006-2022).

|  | per IQR increase ^a^ | Air pollution concentrations (quintiles) | | | | *P* for trend |
| --- | --- | --- | --- | --- | --- | --- |
|  |  | Q1^b^ | Q2^b^ | Q3^b^ | Q4^b^ |  |
| Model 1^a^ | 1.16 (1.11~1.21) | 1.00 | 1.19 (1.08~1.32) | 1.29 (1.17~1.42) | 1.33 (1.21~1.47) | 9.96×10^-10^ |
| Model 2^b^ | 1.13 (1.07~1.18) | 1.00 | 1.12 (1.01~1.25) | 1.21 (1.08~1.34) | 1.25 (1.12~1.40) | 2.62×10^-5^ |

Note: ^a^ Each air pollution concentration was treated as continuous variable and the HR per interquartile range (IQR) increase and its 95%CI were reported.

^b^ Each air pollution concentration was divided into four categories (Q1-Q4), and HR per one quartile increment and its 95%CIs were reported. Q1: the first quartile; Q2: the second quartile; Q3: the third quartile; Q4: the least quartile.

Model 1: adjusted for age and sex.

Model 2: included covariates in model 1 and adjusted for additionally education agree, BMI, drinking status, smoking status, physical activity, healthy diet score, urban residency, TDI, and family history.

# Table S10. Adjusted hazard ratio and 95% confidence interval for air pollution score with the risk of incident dementia by including participants aged <50 years at baseline who were excluded in main analysis (United Kingdom, 2006-2022).

|  | per IQR increase ^a^ | Air pollution concentrations (quintiles) | | | | *P* for trend |
| --- | --- | --- | --- | --- | --- | --- |
|  |  | Q1^b^ | Q2^b^ | Q3^b^ | Q4^b^ |  |
| Model 1^a^ | 1.16 (1.11~1.21) | 1.00 | 1.17 (1.06~1.29) | 1.28 (1.16~1.41) | 1.33 (1.21~1.46) | 3.50×10^-10^ |
| Model 2^b^ | 1.13 (1.08~1.19) | 1.00 | 1.10 (0.99~1.22) | 1.20 (1.08~1.33) | 1.25 (1.12~1.39) | 1.09×10^-5^ |

Note: ^a^ Each air pollution concentration was treated as continuous variable and the HR per interquartile range (IQR) increase and its 95%CI were reported.

^b^ Each air pollution concentration was divided into four categories (Q1-Q4), and HR per one quartile increment and its 95%CI were reported. Q1: the first quartile; Q2: the second quartile; Q3: the third quartile; Q4: the least quartile.

Model 1: adjusted for age and sex.

Model 2: included covariates in model 1 and adjusted for additionally education agree, BMI, drinking status, smoking status, physical activity, healthy diet score, urban residency, TDI, and family history.

# Table S11. Adjusted hazard ratio and 95% confidence interval for air pollution score with the risk of incident dementia among participants aged ≥60 years at baseline (United Kingdom, 2006-2022).

|  | per IQR increase ^a^ | Air pollution concentrations (quintiles) | | | | *P* for trend |
| --- | --- | --- | --- | --- | --- | --- |
|  |  | Q1^b^ | Q2^b^ | Q3^b^ | Q4^b^ |  |
| Model 1^a^ | 1.14 (1.09~1.19) | 1.00 | 1.18 (1.06~1.31) | 1.27 (1.14~1.41) | 1.30 (1.17~1.45) | 9.56×10^-8^ |
| Model 2^b^ | 1.10 (1.05~1.17) | 1.00 | 1.12 (1.00~1.25) | 1.20 (1.06~1.34) | 1.23 (1.09~1.38) | 2.93×10^-4^ |

Note: ^a^ Each air pollution concentration was treated as continuous variable and the HR per interquartile range (IQR) increase and its 95%CI were reported.

^b^ Each air pollution concentration was divided into four categories (Q1-Q4), and HR per one quartile increment and its 95%CI were reported. Q1: the first quartile; Q2: the second quartile; Q3: the third quartile; Q4: the least quartile.

Model 1: adjusted for age and sex.

Model 2: included covariates in model 1 and adjusted for additionally education agree, BMI, drinking status, smoking status, physical activity, healthy diet score, urban residency, TDI, and family history.

# Table S12. Drugs information in the United Kingdom Biobank (United Kingdom, 2006-2022).

| Drug categories | Drugs | Codes |
| --- | --- | --- |
| Anti-hypertensive drugs | Lisinopril | 1140860696 |
|  | Ramipril | 1140860806 |
|  | Bendrofluazide | 1140866122 |
|  | Atenolol | 1140866738 |
|  | Bisoprolol | 1140879760 |
|  | Perindopril | 1140888560 |
|  | Candesartan cilexetil | 1141156836 |
|  | Lisinopril | 1140860696 |
| Statins | Simvastatin | 1140861958 |
|  | Pravastatin | 1140888648 |
|  | Atorvastatin | 1141146234 |
| Insulin | Insulin product | 1140883066 |
|  | Metformin | 1140884600 |

# Table S13. Definitions and sources of information for comorbidities in the United Kingdom Biobank (United Kingdom, 2006-2022).

| Diseases | ICD-9 | ICD-10 |
| --- | --- | --- |
| Stroke | 430, 431, 433, 434, 436 | I60, I61, I63, I64 |
| Hypertension | 401, 4010, 4011, 4019, 402, 4020, 4021, 4029, 403, 4030, 4031, 4039, 404, 4040, 4041, 4049, 405, 4050, 4051, 4059 | I10, I11, I11.0, I11.9, I12, I12.0, I12.9, I13, I13.0, I13.1, I13.2, I13.9, I15, I15.0, I15.1, I15.2, I15.8, I15.9 |
| Atrial fibrillation | 427.31, 427.32 | I48, I48.1, I48.2, I48.3, I48.4, I48.9, K62.1, K62.2, K62.3, K62.4 |
| Cardiovascular disease | 410, 410.0, 410.1, 401.2, 410.3, 410.4, 410.5, 410.6, 410.7, 410.8, 410.9, 410.91, 411, 411.0, 411.1 411.8, 411.89, 411.9, 412, 412,9, 413, 413.1, 413.9, 414, 414.0, 414.8, 414.9, 429.79 | I20, I20.0, I20.1, I20.8, I20.9, I21, I21.0, I21.1, I21.2, I21.3, I21.4, I21.9, I22, I22.0, I22.1, I22.8, I22.9, I23, I23.0, I23.1, I23.2, I23.3, I23.4, I23.5, I23.6, I23.8, I24, I24.0, I24.9, I25, I25.1, I25.2, I25.5, I25.6, I25.8, I25.9 |

Note: ICD: International Classification of Disease.

# Table S14. Adjusted hazard ratio and 95% confidence interval for air pollution score with the risk of incident dementia among participants aged ≥60 years at baseline (United Kingdom, 2006-2022).

|  | per IQR increase ^a^ | Air pollution concentrations (quintiles) | | | | *P* for trend |
| --- | --- | --- | --- | --- | --- | --- |
|  |  | Q1^b^ | Q2^b^ | Q3^b^ | Q4^b^ |  |
| Model 1^a^ | 1.17 (1.10~1.24) | 1.00 | 1.11 (0.96~1.27) | 1.23 (1.07~1.40) | 1.30 (1.13~1.49) | 6.41×10^-5^ |
| Model 2^b^ | 1.12 (1.05~1.20) | 1.00 | 1.03 (0.89~1.19) | 1.11 (0.96~1.29) | 1.16 (0.99~1.34) | 0.045 |

Note: ^a^ Each air pollution concentration was treated as continuous variable and the HR per interquartile range (IQR) increase and its 95%CI were reported.

^b^ Each air pollution concentration was divided into four categories (Q1-Q4), and HR per one quartile increment and its 95%CI were reported. Q1: the first quartile; Q2: the second quartile; Q3: the third quartile; Q4: the least quartile.

Model 1: adjusted for age and sex.

Model 2: included covariates in model 1 and adjusted for additionally education agree, BMI, drinking status, smoking status, physical activity, healthy diet score, urban residency, TDI, and family history.


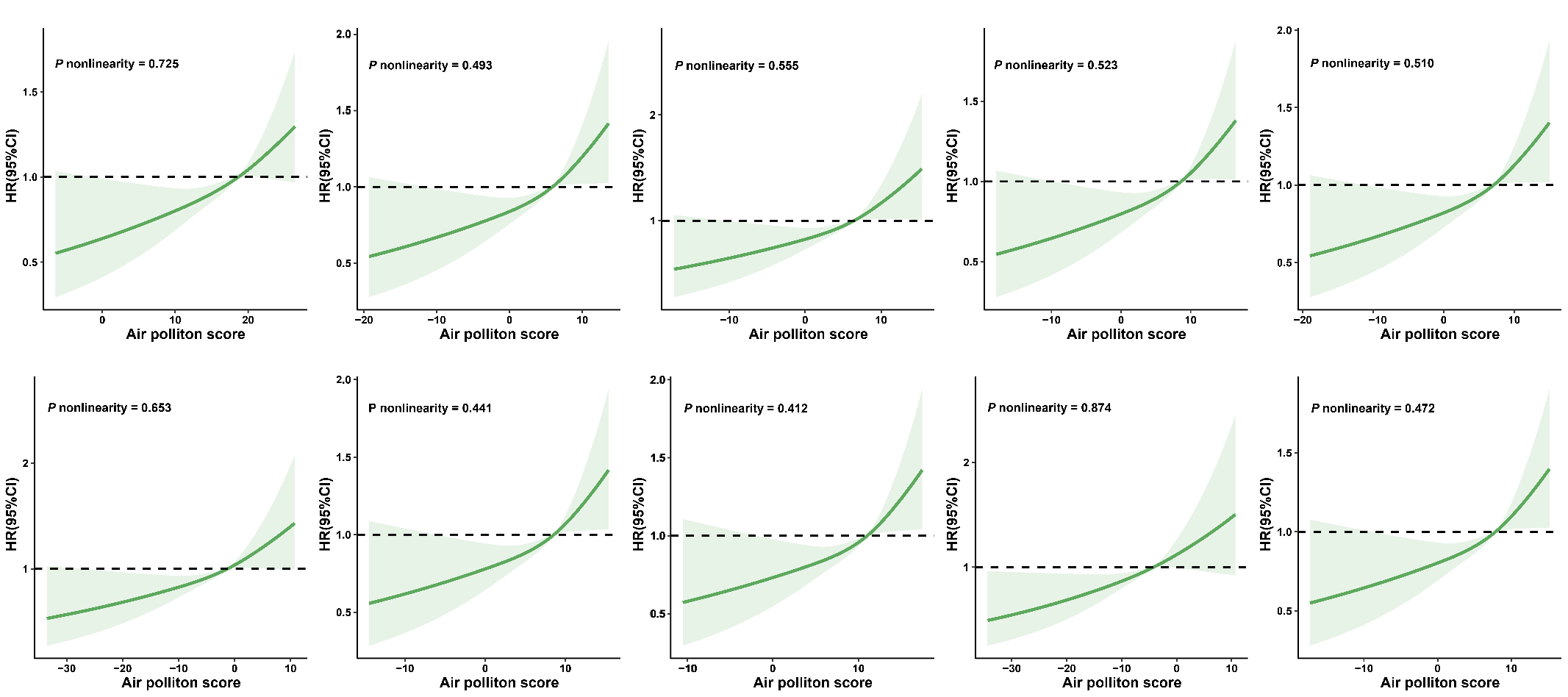


Figure S1. Distribution of air pollution score and restricted cubic spline curves of air pollution score on dementia during the 10-fold cross-validation analysis (United Kingdom, 2006-2022).


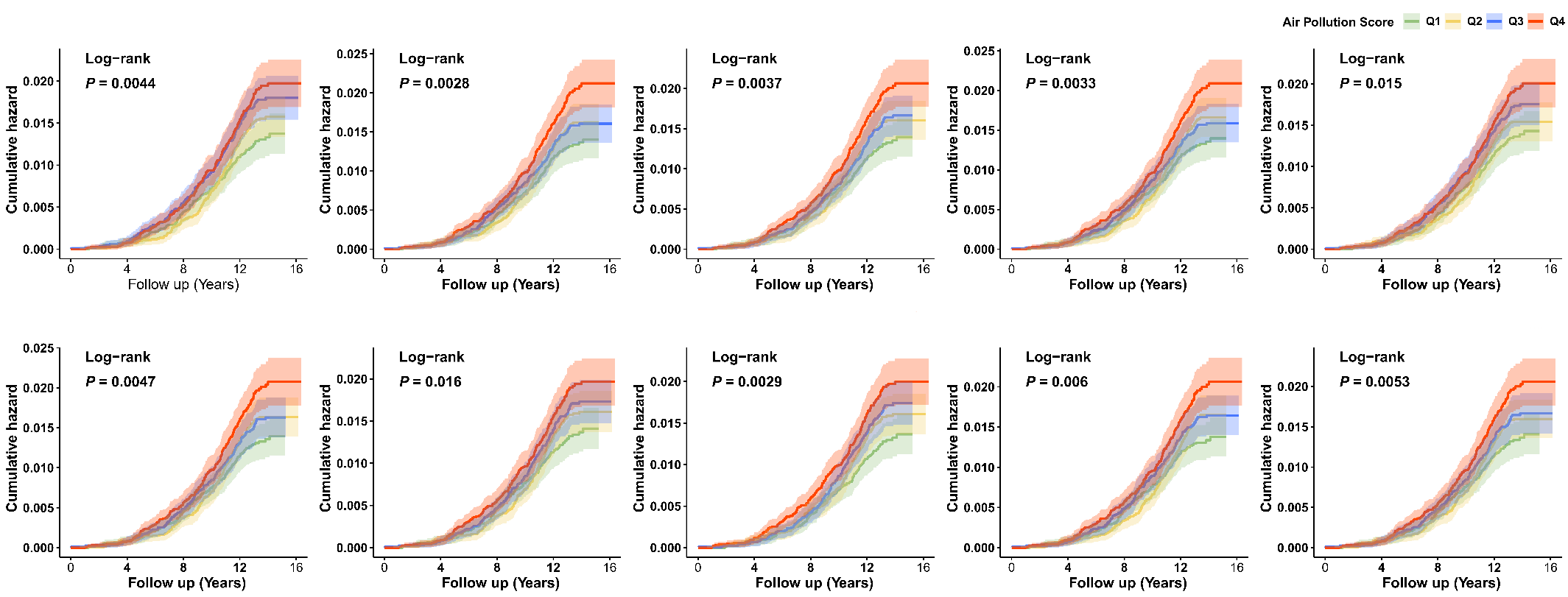


Figure S2. Kaplan-Meier survival analysis of incident dementia according to different quartiles of air pollution score during the 10-fold cross-validation analysis (United Kingdom, 2006-2022).


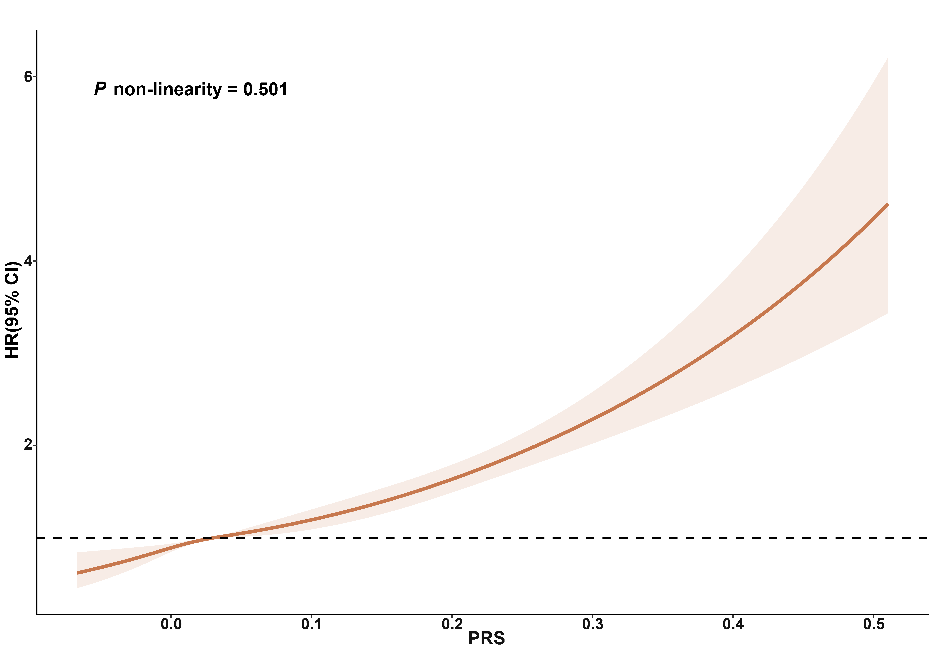


Figure S3. Restricted cubic spline curves of polygenic risk score on incident dementia (United Kingdom, 2006-2022).
